# FoxP3^+^ Regulatory T Cells Attenuate Experimental Necrotizing Enterocolitis

**DOI:** 10.1371/journal.pone.0082963

**Published:** 2013-12-18

**Authors:** Bridgette M. Dingle, Yuying Liu, Nicole Y. Fatheree, Juleen Min, J. Marc Rhoads, Dat Q. Tran

**Affiliations:** Department of Pediatrics, The University of Texas Medical School, Houston, Texas, United States of America; New York University, United States of America

## Abstract

Necrotizing enterocolitis (NEC) results from severe intestinal inflammation in premature infants. FoxP3^+^ regulatory T cells (Tregs) are central to gut homeostasis. While Treg proportions are significantly reduced in the ileums of premature infants with NEC, it is unknown whether they play a critical function in preventing NEC. This study investigated Treg development in newborn rat pups and their role in experimental NEC induction. Utilizing an established rat model of experimental NEC, the ontogeny of T cells and Tregs in newborn pups was characterized by flow cytometry. To investigate the functions of Tregs, newborn pups were given Tregs harvested from adult rats prior to NEC induction to assess clinical improvement and mechanisms of immune regulation. The results revealed that there were few Treg numbers in the terminal ileums of newborn rats and 8-fold reduction after NEC. Adoptive transfer of Tregs significantly improved weight loss, survival from 53% to 88%, and NEC incidence from 87% to 35%. The Tregs modulated the immune response as manifested in reduced CD80 expression on antigen presenting cells and decreased T cell activation within the mesenteric lymph nodes. These findings suggest that while Tregs are present in the intestines, their numbers might be insufficient to dampen the excessive inflammatory state in NEC. Adoptive transfer of Tregs attenuates the severity of NEC by limiting the immune response. Strategies to enhance Tregs have a therapeutic potential in controlling the development of NEC.

## Introduction

Necrotizing enterocolitis (NEC) is a gastrointestinal emergency that results from severe inflammation and necrosis of the bowel wall in premature infants [1]–[3]. It is considered to be a leading cause of morbidity and mortality in the neonatal intensive care unit (NICU). Prematurity may be the most important risk factor for the development of NEC. Among premature infants, the very low birth weight (VLBW<1500 g) group has the highest risk. Despite advances in clinical care and medical technology that have improved the ability to support premature infants, the prevalence of NEC has not decreased [4], [5]. The mortality rate for patients with surgical NEC varies between 20–50% and approaches 100% in infants with panintestinal involvement [6], [7]. For those that survive the surgical intervention, up to 25% develop significant morbidity which includes feeding abnormalities, failure to thrive, intestinal obstruction, short bowel syndrome, and parental nutrition associated intestinal disease.

Although this phenomenon has been described over a century ago, currently the etiology of NEC remains elusive. Since prematurity is a major risk factor, the susceptibility to NEC might be secondary to a developmental process involving immature intestinal integrity and immune regulation. One possible factor that could account for the development of NEC is insufficient FoxP3^+^ regulatory T cells (Tregs) relative to effector cells. Tregs are critical for establishment of immune homeostasis and maintenance of tolerance [8], [9]. In mice, Tregs have a delayed migration out of the thymus relative to conventional T cells [10], [11]. There is also a delayed ontogeny of Tregs in the intestinal tract of mice [12], [13]. Neonatal thymectomy at day 3 of life results in autoimmunity that is preventable by adoptive transfer of Tregs. In humans, patients with immune dysregulation, polyendocrinopathy, enteropathy and X-linked syndrome (IPEX), a rare condition resulting from deficiency in Tregs due to mutations in *FOXP3* gene, develop severe gastrointestinal inflammation characterized by severe villous atrophy and extensive lymphocytic infiltrates of the intestinal mucosa [14]. Interestingly, the gastrointestinal disease is exacerbated when switched from breast milk to cow-based formula, suggesting a dysregulated immune response and lack of tolerance to foreign antigens in the absence of Tregs. The critical role of Tregs in maintaining intestinal homeostasis and preventing colitis and inflammatory bowel disease (IBD) also has been supported by established murine studies [15]–[17]. There is accumulating evidence that Tregs play a vital function in controlling the pathogenesis of IBD in humans [18]–[21]. Therefore, it is plausible that the development, diversity or quantity of Tregs is limited in the intestines of premature infants, resulting in a susceptibility to NEC due to an excessive inflammatory state in this disease. Insufficient quantity or maturation of Tregs could result in dysregulated immune responses to myriad of antigens. A previous study attempted to investigate whether there were inadequate Tregs in the intestines of infants with NEC, but was unable to detect a difference in the quantities of Tregs between preterm and term paraffin-embedded intestinal samples by immunohistochemistry due to technical limitations [22]. Recently, a more detailed study from these investigators was performed using flow cytometry to quantify Tregs in the lamina propria of resected ileums from gestational age-matched infants with and without NEC [23]. They demonstrated that the proportion of Tregs was significantly decreased in premature infants with NEC, suggesting a possible role in controlling excessive inflammation.

Currently, there has been no study to demonstrate a direct function of Tregs in controlling the development and progression of NEC. Insights into the role of Tregs in mediating the pathogenesis of NEC would have an enormous impact in understanding this devastating disease and developing therapeutic strategies for prevention or treatment. Recently, we have demonstrated that feeding probiotic *Lactobacillus reuteri* to newborn rat pups reduced the incidence and severity of experimental NEC in a well-established rat model [24] and that this therapeutic effect correlated with increased frequencies of Tregs in the ileums [25]. To investigate whether Tregs could control the development of NEC, we utilized the same established rat model of experimental NEC [26].

In this study, we initially characterized the ontogeny of T cells and Tregs in newborn pups by flow cytometry. We further determine the functions of Tregs in experimental NEC when newborn pups were given Tregs harvested from adult rats prior to NEC induction to assess clinical improvement and mechanisms of immune regulation.

## Materials and Methods

### Animal model and experimental design

Ethics Statement: Studies were approved by the Animal Welfare Committee of the University of Texas Health Science Center at Houston (# HSC-AWC-10-147).

Experimental groups: All in vivo experiments for NEC model were performed using newborn Sprague-Dawley rat pups (Harlan Laboratories, Indianapolis, IN) weighing 5–6 g. To characterize the changes of Tregs in NEC, NEC group (n = 26) of newborn rats that were separated from their dams, housed in an incubator, fed with formula and induction of NEC, was compared with DAM-fed control group (n = 26) of newborn rats that stayed with their moms. To examine the effects of functional Tregs on newborn rats with NEC, two groups of newborn rats that were separated from their dams, housed in an incubator and fed with formula were intraperitoneally injected (ip) Tregs (NEC+Tregs group, n = 26) or saline (NEC group, n = 38), followed by induction of NEC. Further, to examine whether Tregs attenuated NEC induction by limiting T cell activation, two groups of newborn rats were injected (ip) Tregs (CD4^+^CD25^+^) (n = 8) or Teffs (CD4^+^CD25^−^) (n = 10), respectively, followed by induction of NEC. For ontogeny experiments, newborn rats were euthanized at day of life (DOL) 0–7, 10, 14, and 20 to harvest the spleen, mesenteric lymph node (MLN), thymus, and terminal ileum, n = 6 at each time point.

Experimental NEC model: One day old (DOL1) newborn rats were separated from the dam, housed in an incubator and starved for 12 h before the initiation of formula feeding on day 2 with 100–200 µl of formula using sterile Instech Solomon 22G 35 mm feeding needles, four times daily every 6 hours for 3 days. To induce NEC, rat pups were subjected to 10 min of hypoxia (5% oxygen, 95% nitrogen) three times daily in a Modular Incubator Chamber (Billups-Rothenberg, Del Mar, CA) for 3 days. The formula consisted of 15 g Similac 60/40 (Ross Pediatrics, Columbus, OH) in 75 ml of Esbilac canine milk replacement (Pet-Ag, Hampshire, IL) [26], [27]. Animals were monitored every three hours during 3-day period of study. No analgesia was offered to rats or mice in this experimental NEC model in previously published studies [26], [28]. However, if animals were in pain, demonstrating labored respirations, severe abdominal distension or gastrointestinal bleeding, we euthanized them at this point by peritoneal injection of pentobarbital (FatalPlus) at 1000 mg/kg. Otherwise, pups were euthanized on day 4 after live animal numbers were counted to collect tissues. The % of survival was calculated.

Adult Sprague Dawley rats (2–month old) were euthanized using a CO_2_ chamber followed by cervical dislocation. The spleen, peripheral lymph nodes and MLN were collected to isolate Tregs for adoptive transfer.

### Tissue harvest and NEC evaluation

Following incision of the abdomen, the gastrointestinal tract was carefully removed. The small intestine was evaluated visually for typical gross signs of NEC, such as intestinal distension, wall hemorrhage or necrosis. The terminal ileum was defined as the distal 20% of the length of the small bowel [29]. The terminal 5 cm of small intestine (ileum) was excised. The terminal 1 cm of each sample was formalin fixed and processed by the Cellular and Molecular Morphology Core Lab (the Texas Medical Center Digestive Diseases Center, Houston, TX) and stained with hematoxylin and eosin (H&E) for histological evaluation. The remaining 4 cm of small intestines was used for isolation of lymphocytes. Histological NEC scores were defined as described previously [24], [27]. Briefly, histological scores in the ileum were scored by 2 blinded evaluators on a scale of 0 (normal), 1 (mild, separation of the villous core, without other abnormalities), 2 (moderate, villous core separation, submucosal edema, and epithelial sloughing), and 3 (severe, denudation of epithelium with loss of villi, full-thickness necrosis). The analysis was performed on multiple 5 µm thick sections similar to other published studies [30]–[32]. The final score was based on the worst-appearing microscopic field. Animals with histological scores ≥ 2 were defined as having NEC. Histological scores were only obtained from the tissues of live animals, therefore, N = 20 for NEC group and N = 23 for NEC+Tregs group.

### Tissue Preparation for Flow Cytometry

Single cell suspensions from the spleen, thymus, and total MLN were obtained by gently fragmenting and filtering the tissues through 40 µm cell strainers (BD Biosciences) into MACS buffer consisting of phosphate buffered saline (PBS), 0.5% bovine serum albumin (BSA) (Hyclone Laboratories) and 2 mM EDTA (Lonza). The red blood cells (RBCs) from the spleen were lysed using ACK Lysing Buffer (Quality Biological, Inc.). For the terminal ileum, tissue was incubated for 30 minutes at 37°C in RPMI-1640 (Sigma) complete medium containing collagenase V from clostridium histolyticum (Sigma) at the concentrations of 0.1 mg/mL followed by vigorously vortexing for 1 min. Afterwards, it was dissociated through a 40 µm cell strainer to achieve single cell suspension. Cell count for the terminal ileum was based on this fixed length. For dendritic cell (DC) analysis, the MLN were digested similar to the intestines. Cell count was performed with a hemocytometer and trypan blue exclusion.

### Flow Cytometric Analysis

Cells were surface stained using the following mouse anti-rat antibodies: CD3 (1F4), CD4 (OX-38), CD8a (OX-8), CD25 (OX-39), TCR γδ (V65), CD80 (3H5), CD86 (24F), CD62L (OX-85), CD103 (OX-62), RT1D (OX-17), CD11b/c (OX-42) all from BioLegend and granulocyte marker (HIS48) from eBioscience. Intracellular staining for FoxP3 and Helios was performed with fixation/permeabilization kit per manufacturer protocol (eBioscience) and detected with anti-FoxP3 (FJK-16s, eBioscience) and anti-Helios (22F6, Biolegend). For intracellular staining of IFNγ (DB-1, Biolegend) and IL17A (eBio17B7, eBioscience), the cells were stimulated with 50 ng/mL phorbol 12-myristate 13-acetate (PMA), 1 µg/mL ionomycin and 5 µg/mL brefeldin A (all Sigma-Aldrich) for 5 hours and processed with the same fixation/permeabilization kit. All samples were analyzed with BD FACSCalibur. Data were processed with FlowJo (Tree Star, Inc.).

### Adoptive Treg Immunotherapy

The spleen, peripheral lymph nodes and MLN collected from 7 adult rats were processed sterilely into cell suspension as described above and re-suspended in MACS buffer. The typical cell yield from 7 adult rats was ∼1.5 billion cells. The single cell suspension was labeled with anti-rat CD25 PE Abs (0.2 mg/ml) at 0.03 µl per 10^6^ cells for 30 minutes at 4°C, washed and then incubated with anti-PE beads (Miltenyi Biotecs) at 0.1 µl per 10^6^ cells. The CD25^+^ cells were isolated by a two-step process using the AutoMACS Cell Separator (Miltenyi Biotecs), first with possels followed by possel mode. Starting with the CD25^−^ population, the CD4^+^CD25^−^ T effector cells (Teffs) were isolated by the same method using anti-CD4 PE and anti-PE beads. For tracking the Tregs in vivo, the cells were labeled with 10 µM CFSE using a CellTrace CFSE Kit (Life Technologies) according to manufacturer instructions. The CFSE-labeled Tregs were resuspended in sterile 0.9% sodium chloride solution at a concentration of 10^6^ per 50 µl. Newborn rats were injected intraperitoneally with 50 µl containing 10^6^ Tregs as NEC+Tregs group, or 50 µl containing 10^6^ Teffs as NEC+Teffs, or 50 µl of sterile 0.9% saline as NEC control group before induction of NEC.

### Statistics

Experimental results were expressed as mean ± standard error (SE). The Wilcoxon-Mann Whitney test was used for statistical analysis of all the experiments to determine the differences between groups (GraphPad Prism v6.02 and Stata 11 SE). Chi-square test was used for comparison of Kaplan-Meier survival (%) curves between NEC+Tregs vs. NEC groups. A p-value of <0.05 was considered statistically significant.

## Results

### Frequency of Tregs in experimental necrotizing enterocolitis

**Figure 1:**
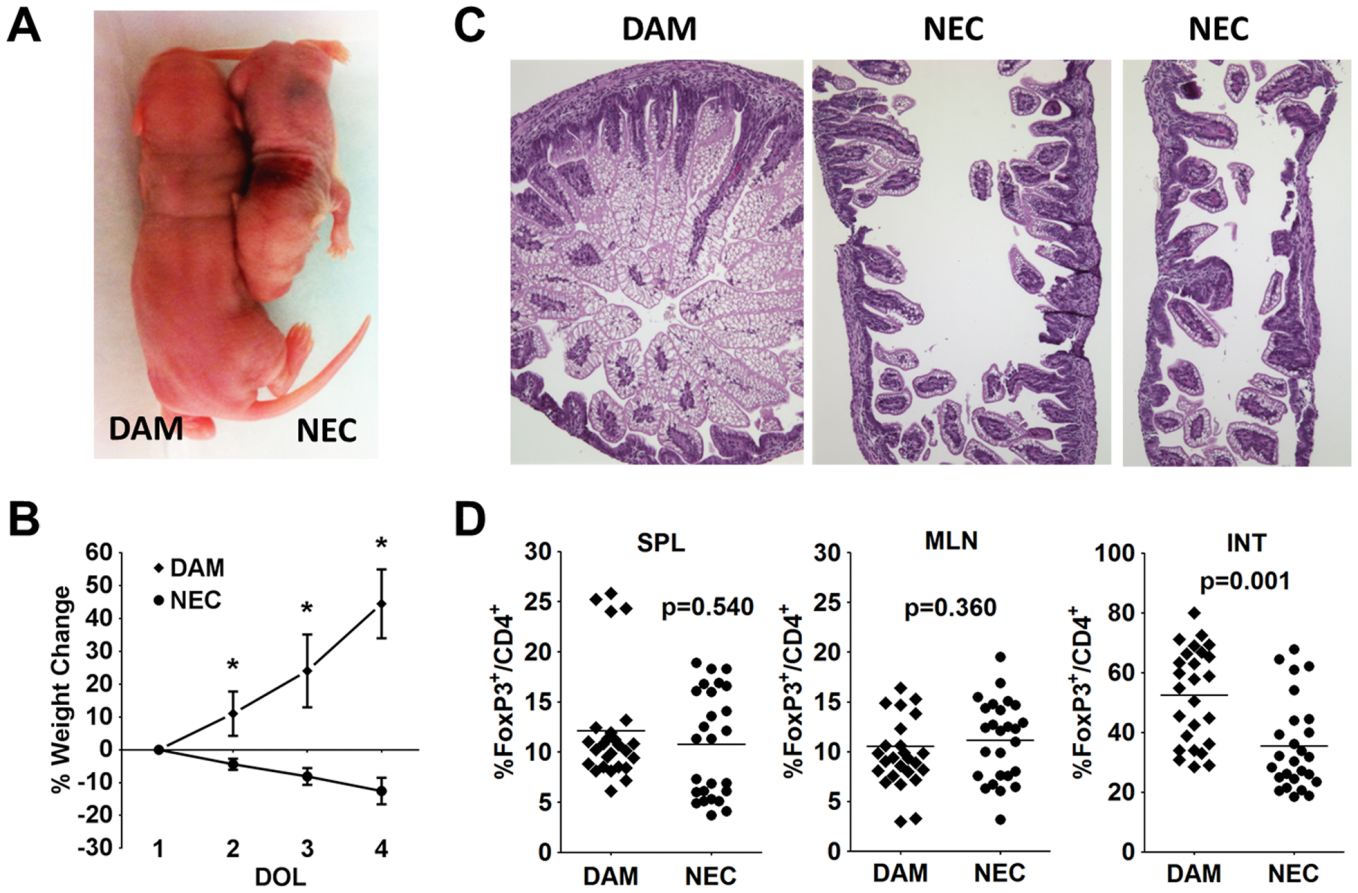
Frequency of Tregs in experimental necrotizing enterocolitis. (A) Representative gross size difference between DAM (left) and NEC (right) rat pups at DOL 4. (B) Weight change for DAM vs. NEC pups for the 3 day experimental time period. Asterisks indicate p<0.001. (C) Representative H&E staining of terminal ileums from DAM and NEC. (D) Percentages of FoxP3^+^ Tregs within CD4^+^ T cells between DAM and NEC within the spleens (SPL), mesenteric lymph nodes (MLN) and terminal ileums (INT). Data are combined 3 independent experiments with n = 26 for each group.

Multiple factors have been implicated as triggers initiating NEC. These triggers include hypoxia, hemodynamic instability, formula osmolality, infections and hypothermia. In this rat model of experimental NEC, the optimal induction of NEC requires a combination of formula feeding and hypoxia. During the 3 day of induction, there was a dramatic weight loss in the NEC group compared to the dam-fed group, based on size and weight (Fig. 1A,B). While the average starting weight was around 6 g for both groups, the dam-fed pups gained an average of 45% on day 4. In the NEC group, there was an average weight loss of 4% on day of life (DOL) 2, 8% on DOL 3 and 12% on DOL 4. The dam-fed pups had 100% survival over the 3 days, while the NEC group had a survival rate that dropped to 95% on DOL 3 and 58% on DOL 4 (p<0.05). All pups in the dam-fed group received NEC scores of 0, indicating normal ileal histology (Fig. 1C). In the NEC group, there was around 75% incidence of NEC development, with many receiving a maximum score of 3, indicating severe villus denudation and full thickness necrosis (Fig. 1C). We next examined whether the frequencies of Tregs were perturbed in the NEC group. While the percentages of FoxP3^+^ Tregs within CD4^+^ T cells were similar in the spleen and MLN of DAM versus NEC, there was a significant reduction from an average of 52% Tregs within the terminal ileums of DAM to 35% Tregs of NEC group (Fig. 1D, S1). This finding is consistent with our previous report [25]. This observation prompted our hypothesis that there might be insufficient Treg numbers in the newborn rats, resulting in the susceptibility to NEC induction.

### The ontogeny of T cells and Tregs in dam fed control rat pups

**Figure 2:**
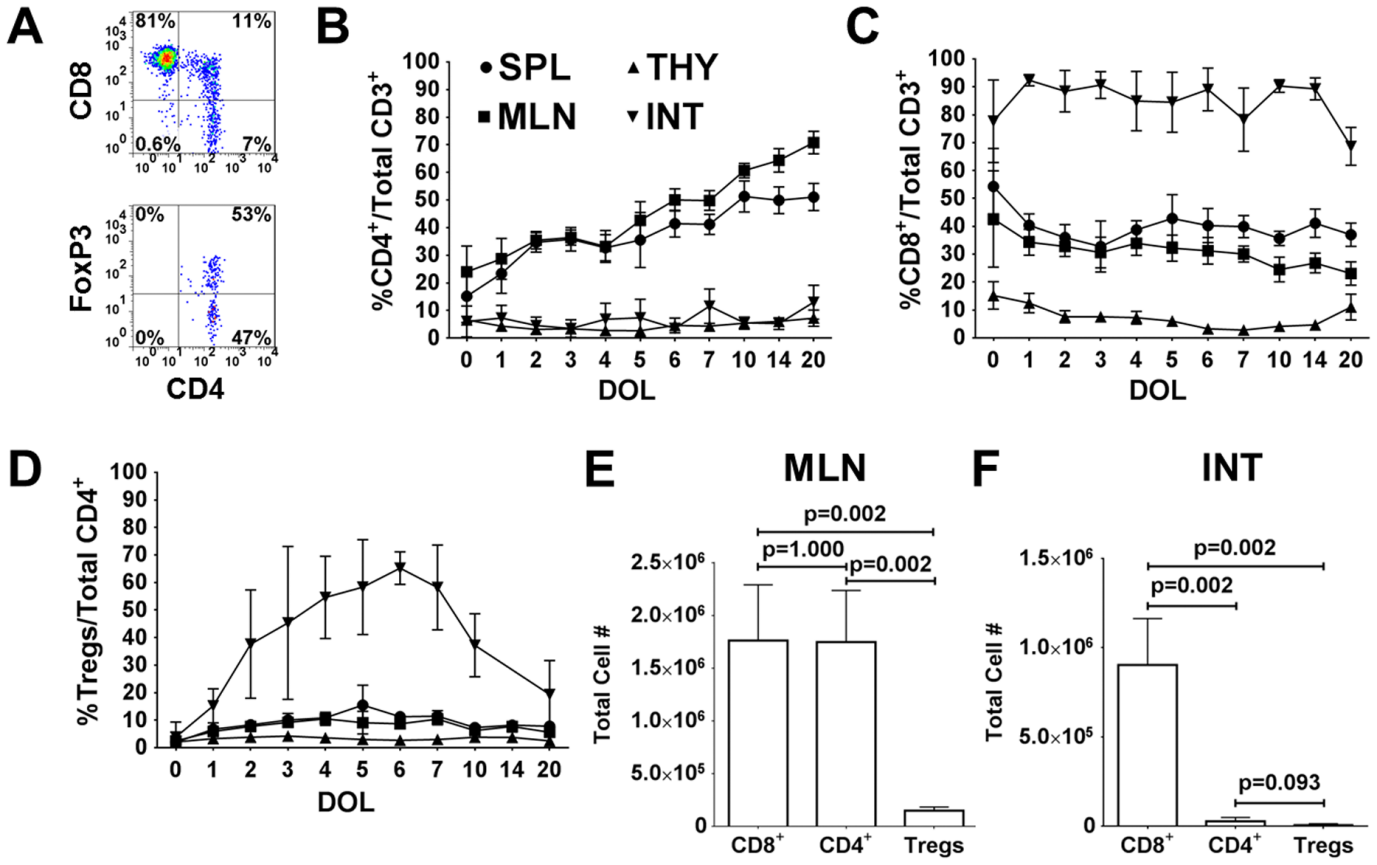
The ontogeny of T cells and Tregs in dam fed control rat pups. (A) Representative flow cytometric plots from terminal ileum on DOL 4 with initial gating on CD3^+^ T cells to determine the percentages of CD4^+^ and CD8^+^ T cells (top plot) and FoxP3^+^ Tregs within CD8^−^CD4^+^ T cells (bottom plot). Frequency of (B) CD4^+^, (C) CD8^+^ and (D) Tregs within the spleen (SPL), thymus (THY), mesenteric lymph nodes (MLN) and terminal ileum (INT). Absolute cell counts of CD8^+^, CD4^+^, and Tregs in the (E) MLN and (F) terminal ileums on DOL 4. Data are derived from n = 6–16 pups at each time point for B–D and n = 6 for E–F.

The normal ontogeny of T cells and Tregs was investigated in newborn rat in order to understand the role of Tregs in NEC. T cell distributions were assessed in the thymus, spleen, MLN and terminal ileum throughout the first 20 days of life. Figure 2A is a flow cytometric representation of an intestinal sample from DOL 4, showing the percentage of CD4^+^ and CD8^+^ within CD3^+^ T cells and the percentage of FoxP3^+^ within CD4^+^CD8^−^ T cells. The average percentage of CD4^+^CD8^−^ T cells remained steady below 15% in the thymus and terminal ileum (Fig. 2B). There was a progressive increase in the percentage of CD4^+^ within the spleen and MLN, beginning with around 15% and 25% at DOL 0, respectively. In the thymus, the CD8^+^CD4^−^ T cells declined from 15% on DOL 0 to below 10% (Fig. 2C). The percentage of CD8^+^ in the spleen and MLN was higher than that of CD4^+^ during the first few days of life and then became lower than CD4^+^ cells. However, the most remarkable finding was the disproportionately greater percentage and number of CD8^+^ compared to CD4^+^ within the terminal ileum (Fig. 2C,F). Over 75% of CD3^+^ T cells were CD8^+^ in the terminal ileum and remained at this high level throughout the 20 days of life. Since we wanted to measure total cellular content within the intestines, the majority of CD8^+^ T cells (>75% CD103^+^) most likely represented intraepithelial lymphocytes (IEL) and not just lamina propria cells. In contrast, the study by Weitkamp et al. showed that in the lamina propria of human infant intestines there were 3–4 times as many CD4^+^ as CD8^+^ cells [23].

Tregs were quantified based on the expression of FoxP3 within CD4^+^CD8^−^ T cells (Fig. 2D). On DOL 0, the percentages of Tregs within all tissues were below 5%. The percentages of Tregs increased to 5–15% within the spleen and MLN, which were similar to reported percentages in mice [33]. Treg percentages in the thymus remained stable below 5%. Interestingly, the frequency of Tregs climbed rapidly in the terminal ileum, peaking at over 60% on DOL 6 and then progressively decreasing to adult levels. Within the Treg population for the first 14 days of life, there was a progressive reduction in the Treg subset that expressed the transcription factor Helios (Fig. S2A, B, C) [34], [35]. The greatest reduction occurred in the intestinal compartment, supporting the evidence of gut environment preferential extrathymic Treg development from naïve T cells [36].

While over 40% of CD4^+^ T cells were Tregs starting on DOL 2, it should be noted that the quantitative numbers of CD4^+^ and Tregs were remarkably low relative to CD8^+^ cells in the terminal ileum (Fig. 2F). The average number of CD8^+^ in the terminal ileum on DOL 4 was 9×10^5^, which was over 30 times greater than CD4^+^ (2.8×10^4^) and over 100 times greater than Tregs (8,800). In the MLN, the average number of CD8^+^ (1.76×10^6^) and CD4^+^ (1.75×10^6^) was similar, while the number of Tregs (1.49×10^5^) was 12 times less than CD8^+^ or CD4^+^ cells (Fig. 2E). These findings support the hypothesis that while Tregs are present early in postnatal life, their numbers might be insufficient to control perturbed immune responses. In premature infants, the quantity of Tregs might be necessary to suppress an exaggerated, unchecked immune response to foreign antigens originating from food and organisms within the immature intestinal lumen.

### Treg immunotherapy reduced the severity and improved the survival of NEC

**Figure 3:**
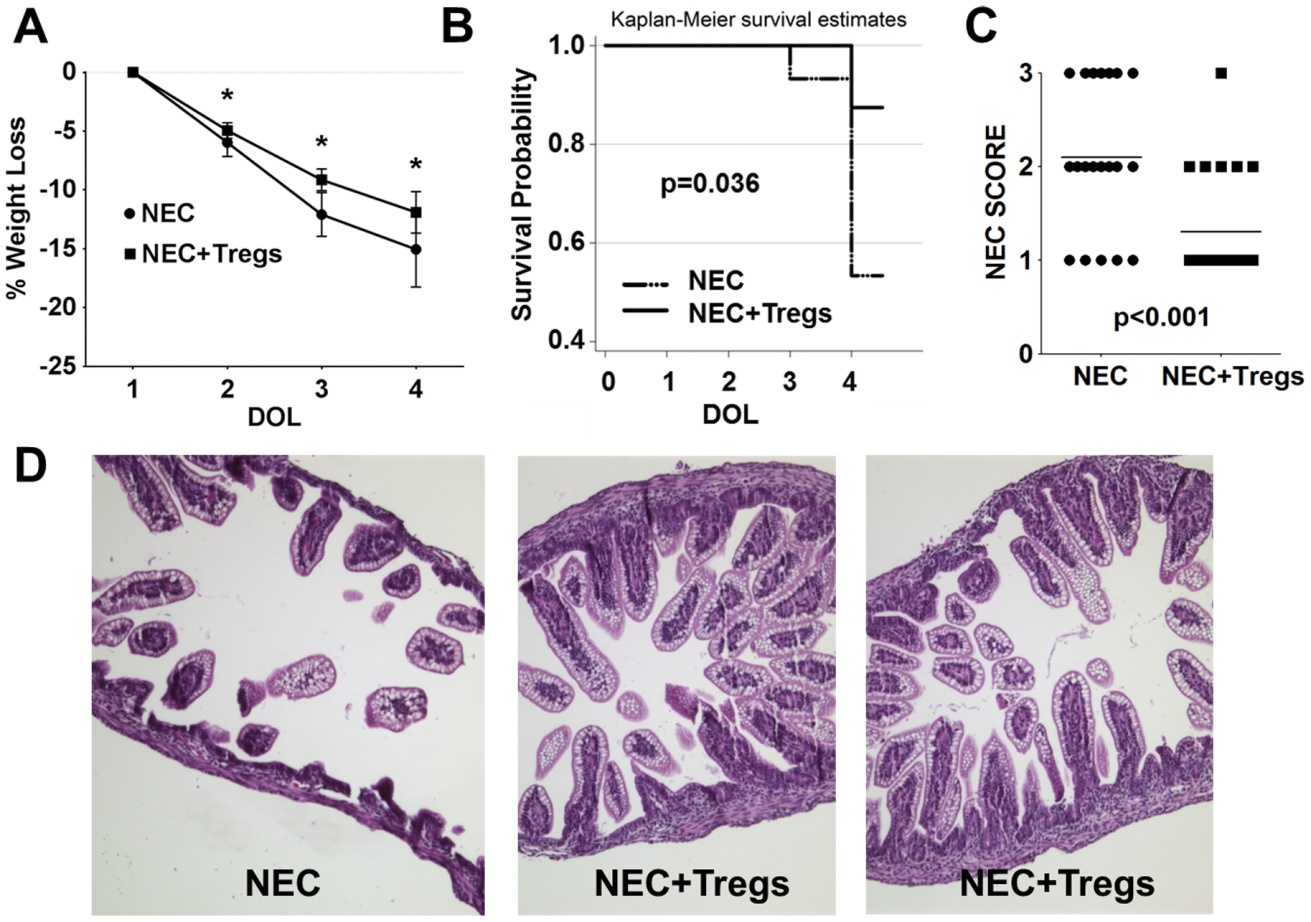
Treg immunotherapy reduced the severity and improved the survival of NEC. (A) Percent of total body weight loss during the 3 day experimental time period between NEC vs. NEC+Tregs groups. Asterisks indicate p<0.05. (B) Kaplan-Meier survival curve for NEC (dotted line) vs. NEC+Tregs (solid line) during the 3 day experimental time period. (C) NEC score from terminal ileal samples of NEC (n = 20) vs. NEC+Tregs (n = 23). (D) Representative H&E staining of terminal ileums from NEC and NEC+Tregs groups. Data are combined three independent experiments with a total starting number of n = 38 for NEC and n = 26 for NEC+Tregs groups.

Based on our hypothesis, we investigated whether Tregs could play a critical role in regulating the development and pathogenesis of NEC in these pups. To address this question, we performed adoptive Treg immunotherapy using Tregs harvested from adult female rats because of the Treg quantity needed. FoxP3 purity in the transferred Tregs was >90% (Fig. S2D). In the NEC+Tregs group, there was a difference in the gross appearance of the pups on DOL 4 after receiving 1×10^6^ Tregs on DOL 1. The pups in the NEC group were smaller in size and more ill-appearing. The gross appearance of the intestines showed necrotic bowel and visible pneumatosis (data not shown). Consistent with these findings, there was a greater weight loss in the NEC group, particularly on DOL 3 and 4 (Fig. 3A). On DOL 3 the mean weight loss was 9.6% in the NEC+Tregs and 12% in the NEC group (p = 0.001). By DOL 4, the NEC group continued to have greater weight loss at 15% compared to 12% for NEC+Tregs (p = 0.008). However, most impressive was the dramatic improvement in survival, NEC incidence and NEC score. On DOL 3, there was 100% survival in the NEC+Tregs and 93% in the NEC group (Fig. 3B). By DOL 4, there was an 88% survival rate for the NEC+Tregs versus a 53% for the NEC group (p = 0.036). Of the 20 out of 38 pups in the NEC group from three independent experiments that we were able to freshly harvest the terminal ileums since the remaining died overnight, many had histological changes correlating with a severe NEC score of 3 (Fig. 3C,D) and a NEC incidence of 75% (87% including the 18 dead pups). In the NEC+Tregs group, the majority had preservation of the villus structure and lamina propia, indicating a NEC score of 1 and a NEC incidence of 26% (35% including the 3 dead pups). Due to the difficulty of feeding these tiny pups and the formula given, maintaining sufficient nutrition was a major issue as reflected by the weight lost, even in the NEC+Tregs group. Therefore, it was difficult to study survival beyond DOL4 because of feeding issues.

**Figure 4:**
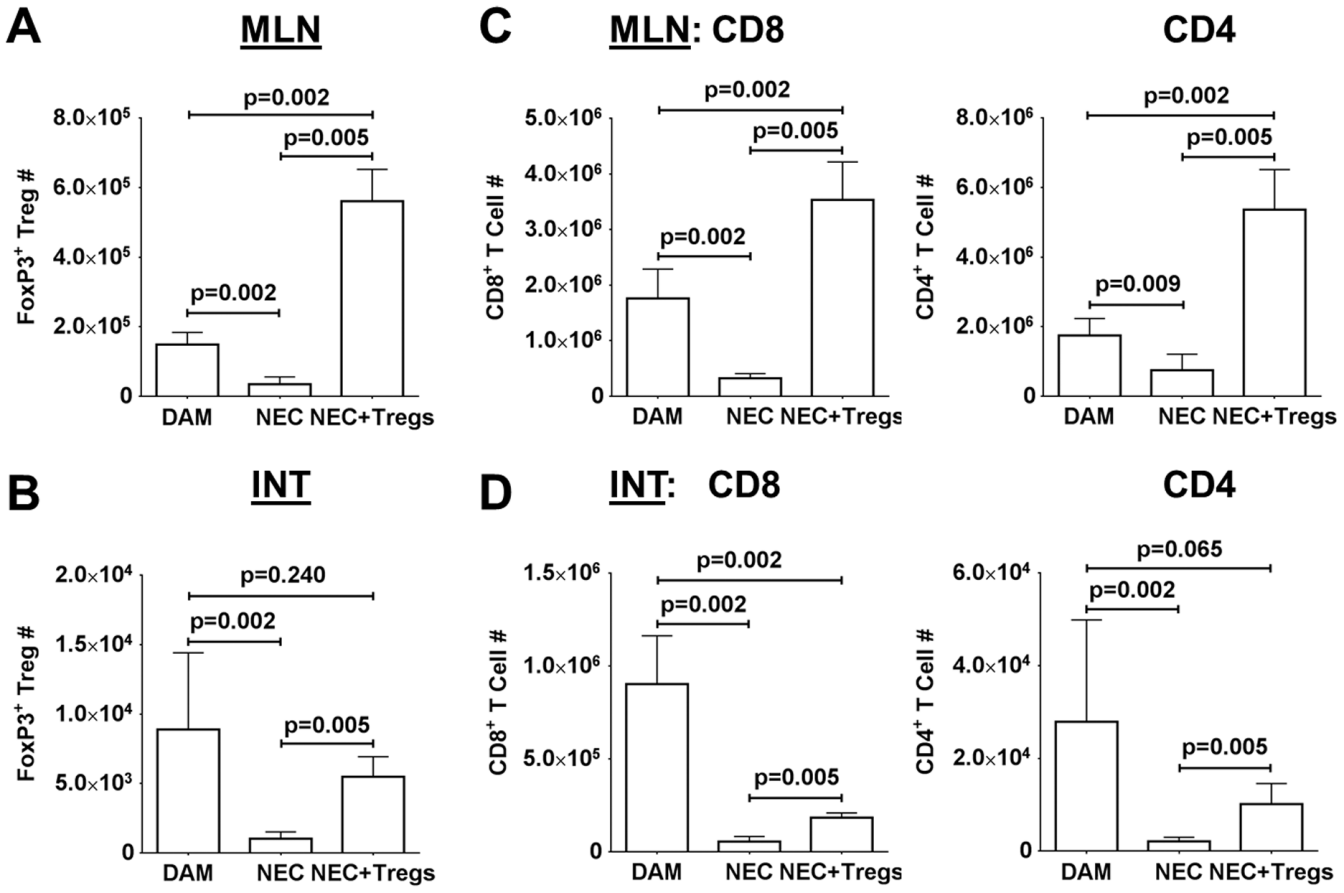
Cellularity after Treg adoptive immunotherapy. Quantitative Treg cell counts within the (A) MLN and (B) INT among the DAM and surviving pups in the NEC and NEC+Tregs groups. Quantitative numbers of CD8^+^ and CD4^+^ T cells within the (C) MLN and (D) terminal ileums. Data are combined three independent experiments with DAM = 26, NEC = 20 and NEC+Tregs = 23.

Since the exogenous Tregs were labeled with CFSE, we could track their migration. Upon examination, the exogenous Tregs were localized predominately in the spleens and MLN but not in the terminal ileums (Fig. S3A). While the percentages of Tregs were similar between DAM and NEC in the MLN (Fig. S3B), there was a significant decrease in cell numbers in the NEC group, from an average of 1.5×10^5^ in the DAM to 3.4×10^4^ in the NEC (Fig. 4A). Interestingly, there was a disproportionate increase in the Treg number (mean 5.6×10^5^) within the MLN of NEC+Tregs group. Since the exogenous Tregs only accounted for around 20% of the FoxP3^+^ cells, this finding suggested that the exogenous Tregs enhanced the migration of endogenous Tregs into the MLN. In the terminal ileums, the Treg numbers between the DAM (8.9×10^3^) and NEC+Tregs (5.5×10^3^) groups were similar not because of a reconstitution by the exogenous Tregs, but because of a greater preservation in normal cellularity and gut integrity (Fig. 3D, 4B). Consistent with the decrease percentages of Tregs in the terminal ileums of the NEC group, there was also a significant reduction in cell numbers (1×10^3^) as compared to DAM group (Fig. 4B).

We next examined the effect of Treg treatment on the cellularity within the MLN and terminal ileums. Based on our flow cytometric analysis and frequency in each subset, we calculated the cell number for CD8^+^ and CD4^+^ T cells (Fig. S3C). When we quantified the cellular composition among the three groups, we noted a significant increase in CD8^+^ and CD4^+^ T cells within the MLN of NEC+Tregs (Fig. 4C). In contrast, the CD8^+^ and CD4^+^ T cells were significantly less in the NEC as compared to DAM group. In the terminal ileums of the NEC group, there was a dramatic reduction in these cells due to end-stage inflammation, necrosis and tissue destruction (Figure 1C, 3D, 4D). However, there was a notable improvement in the total cellularity and T cells in the terminal ileums of NEC+Tregs as compared to the NEC group. The improved outcome with Treg immunotherapy indicates that Tregs can blunt NEC progression and supports the hypothesis that a contributing factor to the pathogenesis of NEC might be due to insufficient Treg numbers or maturation to regulate immune responses and inflammation.

### Tregs attenuated NEC induction by limiting T cell activation

**Figure 5:**
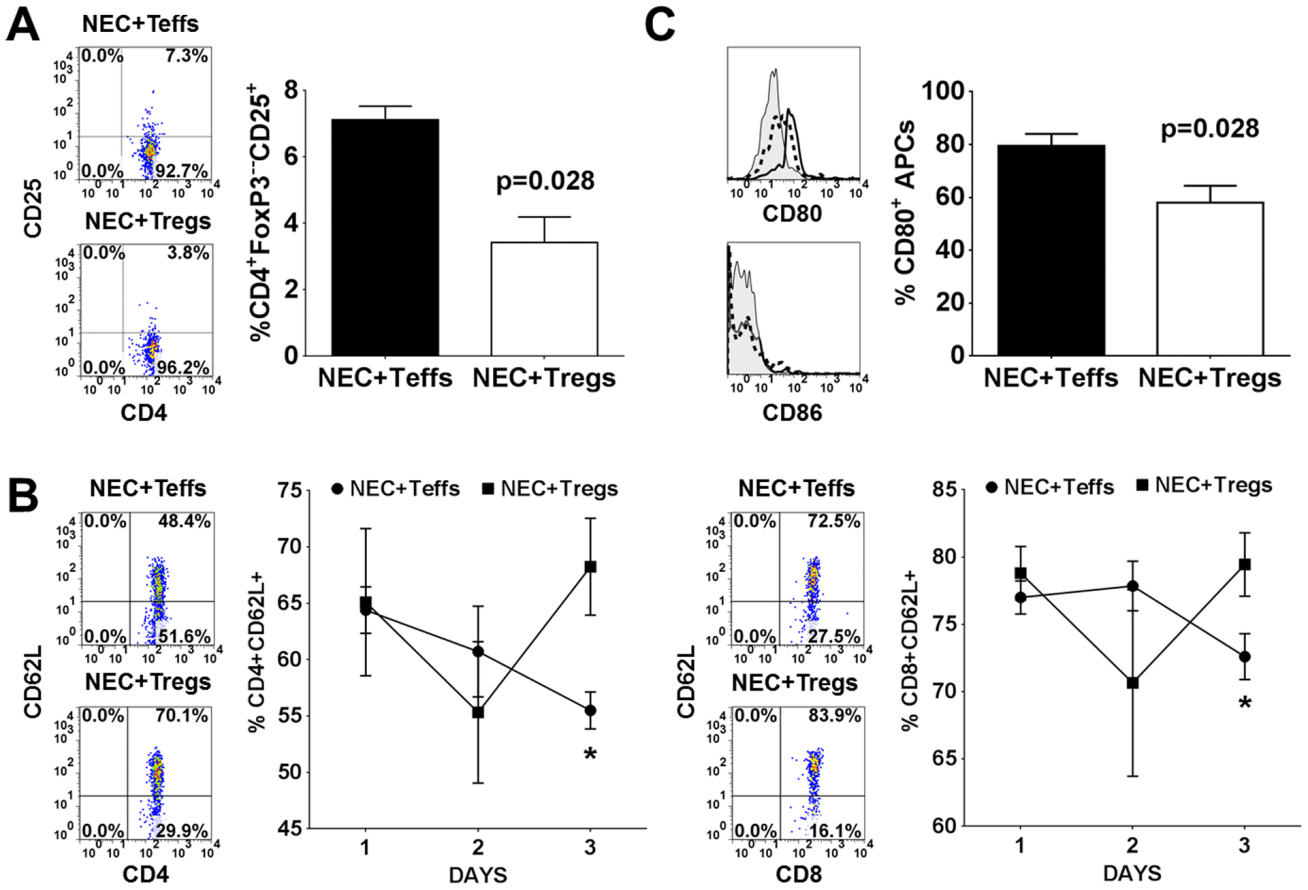
Tregs attenuated NEC induction by limiting T cell activation. (A) Representative FACS plots of CD25 on CD4^+^FoxP3^−^ T cells and total percentages within the MLN at day 3 of NEC induction between NEC+Teffs (n = 10) and NEC+Tregs (n = 8) groups. (B) Representative FACS plots of CD62L expression on CD4^+^ and CD8^+^ T cells at day 3 of NEC induction and the percentages of CD62L^+^ over the 3 day course within the MLN of NEC+Teffs (n = 5, 4, 10 for day 1, 2, 3 respectively) and NEC+Tregs (n = 5, 4, 8 for day 1, 2, 3 respectively). Asterisks indicate p<0.05. (C) Representative histograms of CD80 and CD86 expression on CD11b/c^+^RT1D^−^ (shaded) and CD11b/c^+^RT1D^+^ cells from NEC+Teffs (solid) and NEC+Tregs (dashed) groups. Bar graph is cumulative data of CD80 expression on CD11b/c^+^RT1D^+^ cells from NEC+Teffs (n = 10) and NEC+Tregs (n = 8).

To investigate the mechanism of Treg mediated suppression of NEC severity, we examined the level of immune activation. Multiple studies have shown that Tregs suppress immune responses by down-regulating CD80 and CD86 expression on DCs, which are critical costimulatory molecules involved in T cell activation [37]–[40]. Therefore, we repeated the experiments to examine the level of immune activation between the NEC+Teffs (CD4^+^CD25^−^) and NEC+Tregs (CD4^+^CD25^+^) groups. On the last day of NEC induction, we measured the level of CD25 and CD62L expression on CD4^+^FoxP3^−^ within the MLN. We observed a significant reduction in CD25 expression, an activation marker, within the NEC+Tregs group (Fig. 5A). In the NEC+Teffs group, there was a significant reduction in CD4^+^CD62L^+^ and CD8^+^CD62L^+^ T cells by day 3 of NEC (Fig. 5B). CD62L, also known as L-selectin, is expressed on naïve T cells. It allows for homing to high endothelial venules and migration into peripheral lymph nodes. It is rapidly shed from the lymphocytes upon activation. The lower frequency of CD62L^+^ T cells in the NEC group is consistent with our observation of reduced cell numbers in the MLN (Fig. 4C). These results suggested that Tregs might be targeting APCs to limit T cell activation. Given the limited available reagents to phenotype the APCs, we were only able to assess the APCs based on their expression of CD11b/c and MHC class II (RT1D). Within the CD11b/c^+^ cells localized in the MLN, around 20–30% are granulocytes, <5% expressed CD103, and 8–18% expressed RT1D (Fig. S4). Examination of CD11b/c^+^RT1D^+^ APCs (∼25–60% granulocytes) in the MLN of NEC+Teffs group revealed that an average of 80% expressed CD80 while only 58% in the NEC+Tregs group (Fig. 5C). There was no appreciable expression of CD86 and no differences in the level of CD54 (data not shown) between the two groups. Since CD80 could be constitutively expressed on APCs, the decreased expression in the NEC+Tregs group could be a down-regulation by the Tregs. This decreased CD80 expression correlated with the decreased T cell activation in the NEC+Tregs group. We are perplexed by the absence of significant expression of CD86. Given the lack of reagents specific to rats, we were unable to distinguish and characterize dendritic cells. Overall, these results demonstrate that the adoptively transferred Tregs attenuate the severity of NEC by modulating the immune activation as manifested in reduced expression of CD25 on T cells and CD80 on APCs. The increase in cell numbers and frequencies of CD62L^+^ T cells in the MLN of the Treg-treated group suggests an accumulation of these naïve cells due to the immune suppression by the Tregs to limit their activation and maturation. However, in this rat model as well as NEC in human infants, the pathogenesis is multifactorial. Tregs appear to play a contributory role in reducing the inflammation but not a critical factor in NEC development.

## Discussion

The function of Tregs is to actively participate in the regulation of immune responses and the maintenance of immune tolerance and homeostasis. In murine studies, Tregs have been shown to play an active role in preventing the development of multiple immune-mediated conditions such as EAE, colitis, gastritis, arthritis and graft-versus-host disease (GVHD) [41]–[43]. Because of these preclinical studies, there is a strong focus in developing Treg immunotherapy to prevent and treat these chronic, debilitating conditions [44], [45]. While advances in medical technologies and treatments continue to enhance our ability to save extremely preterm infants, their long-term survival could be limited by the development of NEC. Much investment has been focused on understanding the pathogenesis of NEC. It has been difficult to interrogate the function of Tregs in the pathogenesis of NEC in premature infants. The study by Weitkamp et al. provided the first insight that the proportion of Tregs was significantly reduced in the ileums of premature infants with NEC [23]. However, it is unknown whether Tregs are responsible for regulating the development of NEC. To provide the first preclinical study to address this question, we investigated the role of Tregs in the development of NEC by utilizing a well-studied rat model of experimental necrotizing enterocolitis. It should be noted that no animal model reproduces all the features of human NEC [46]. Key features of various models include cow milk formula feeding, hypoxia, prematurity, parenteral nutrition, stress, and suboptimal nutrition. In support of the human study, the rat pups with NEC exhibited a significant decrease in Tregs in the terminal ileums and the MLN [23]. To provide evidence whether the quantity of Tregs impacted the development of NEC, we demonstrated that adoptive Treg immunotherapy attenuated the severity of NEC. The Tregs used in the immunotherapy were harvested from adult rats which would contain a more mature and memory population. It is unknown whether naïve Tregs obtained from newborn pups would be as efficacious. Furthermore, in this rat model of NEC that occurred over a 3 day period, the Tregs attenuated but did not abrogate its development. Similar to human NEC, this murine model is complicated by many factors including the difficulty in feeding and providing nutrition. This outcome indicates that while Tregs play an important role in regulating inflammation, they are not the single entity that can prevent NEC. There are other factors including nutrition, maturity and intestinal barrier that influence the survival of the pups. While our data suggest that increasing the number of Tregs via cell therapy can mitigate but not prevent the development of NEC, it remains unclear whether the ontogeny of Tregs is delayed in newborn and particularly premature birth. While Tregs appear to play a role in the pathogenesis of human NEC, the mechanisms and etiologies are quite complex and multifactorial. Several human studies have not found any evidence for a delayed ontogeny of Tregs. In human fetal tissues compared to term infants, the proportions of Tregs were in fact higher [47], [48]. In addition, pre- and postnatal development does not appear to correlate with intestinal Treg proportions [22], [23]. In the Weitkamp et al. study, the total number of Tregs, CD4 or CD8 cells from the lamina propria of the ileum was similar, while the Treg/CD4 and Treg/CD8 ratios were significantly lower in patients with NEC compared to controls [23]. In contrast, our murine study showed that there were significantly higher frequencies of Tregs within the DAM compared to the NEC group. One possible explanation was that we analyzed Tregs from total digested terminal ileums and not just the lamina propria. Another possible explanation might be from different factors such as the more sterile environment of the rats and the mechanisms involved in the induction of NEC. Nevertheless, our results established for the first time the direct supportive function of Tregs in regulating the myriad of immune responses at the forefront of an immature, developing intestinal barrier. In their absence or insufficient number, the immature intestines of premature infants are at high risk of an excessive inflammatory response to external insults that could result in the development of NEC.

The novelty and clinical implication of our work is that adoptive transfer of Tregs has the potential to mitigate the severity of NEC. We have shown that the transfer of Tregs prior to NEC induction improves total body weight loss, NEC severity and survival. At the cellular level, we show that the Treg immunotherapy modulates the immune response in the MLN, which is a regional drainage lymphoid organ for the intestine. By controlling the level of immune activation, the Tregs could limit the numbers of destructive T cells from migrating into the tissues to contribute to an unregulated inflammatory response [49]. This mechanism of immune regulation appears to involve an interaction with APCs, although further studies are needed to characterize in greater details. Interestingly, we were only able to detect a difference in the expression of CD80. One possible explanation could be the maturity level of the APCs in those 3–4 day old pups. In an in vitro experiment using human monocyte-derived DCs, one study showed that immature DCs at 96 h expressed high level of CD80 but only a small percentage of cells possessed low level of CD86 [50]. It is well recognized that CD80 and CD86 have differential role and expression. The same study depicted that human Tregs, known to express high level of CTLA4 (CD152), appeared to mediate their suppressive function by preferential interaction of CD152 with CD80 to impair DC functions. A biophysical study examining the interaction properties of CD80/CD86 with CD28/CD152 revealed that CD80 has at least 100-fold greater binding affinity to CD152 over CD28 engagement [51]. Another evidence of the differential function of CD80/CD86 was a study demonstrating that CD80^−/−^ mice had improved survival in a cecal ligation and puncture sepsis model when compared to wild-type or CD86^−/−^ mice [52]. This outcome implies that CD80 signaling plays a dominant role in triggering an innate, inflammatory immune response. While there are multiple other mechanisms including induction of tolerogenic DCs [53], our study at this stage could only reveal a correlation between improvement in NEC with Treg treatment and reduction in immune activation as represented by decreased expression of CD25 and CD80 and increased numbers of CD62L^+^ naïve T cells. Because of the limitations of this rat model, particularly with the availability of reagents, we are moving into a mouse model in order to examine in greater details these mechanisms.

Given the high morbidity and mortality of NEC and the promising outcomes of our study and others, the use of autologous expanded cord blood Tregs is an attractive cellular immunotherapy for the treatment or prevention of this condition. However, it is unclear whether autologous expanded cord blood Tregs would demonstrate efficacy. Future studies are needed to investigate the efficacy and potency of naïve Tregs from cord blood for the treatment or prevention of NEC. Moreover, we do not know the potentially undesired effects of Treg immunotherapy, such as suppression of immune responses to infections, particularly in the preterm infants. Long-term studies in animal models are needed to test the safety and tolerability of Treg therapy. In conclusion, our study is the first to demonstrate in a preclinical rat model of experimental NEC the therapeutic potential of Treg immunotherapy in controlling the severity of NEC. Moreover, our findings further solidify the critical function of Tregs in regulating intestinal homeostasis and their role in the pathogenesis of NEC. Therefore any therapies that could enhance Treg development, migration or induction could have the potential to treat or prevent this debilitating disease.

## Supporting Information

Figure S1 **Representative FACS plots from three independent experiments showing percentages of Tregs in DOL 4 pups from DAM and NEC groups based on FoxP3 expression within CD4^+^CD8^−^ T cells from the spleen (SPL), mesenteric lymph nodes (MLN), and terminal ileum (INT).**
(TIF)

Figure S2 **Frequency of Helios^+^ Treg subset during first 14 days of life in newborn rats showing (A) the expression and correlation of FoxP3 and Helios within CD4^+^ T cells, (B) the percentage of Helios within CD4^+^FoxP3^+^ Tregs and (C) the distribution of Helios^+^ subset within FoxP3^+^ Tregs in the spleen (SPL), mesenteric lymph nodes (MLN), thymus (THY) and terminal ileum (INT). (D) Post-sort FoxP3 purity in the CD4^+^CD25^+^ Tregs and the CD4^+^CD25^−^ non-Treg control from three independent experiments.**
(TIF)

Figure S3 **Representative FACS plots showing (A) the localization of exogenous CFSE-labeled Tregs within the spleen (SPL), MLN and INT, gating on CD4^+^FoxP3^+^ cells, (B) the percentage of Tregs within CD4^+^CD8^−^ T cells from MLN and INT of DOL 4 pups in DAM and the surviving NEC and NEC+Tregs groups, and (C) the percentages of CD4 and CD8 within CD3+ T cells from MLN and INT of DOL 4 pups in DAM and the surviving NEC and NEC+Tregs groups used to calculate cell numbers.**
(TIF)

Figure S4 **(A) Representative FACS plots depicting the percentages of CD11b/c, RT1D, granulocytes and CD103 cells from MLN.** (B) Representative histograms of CD80 and CD86 expression on CD11b/c^+^RT1D^−^ (shaded) and CD11b/c^+^RT1D^+^ cells from NEC+Teffs (solid) and NEC+Tregs (dashed) groups. Bar graph is cumulative data of CD80 expression on CD11b/c^+^RT1D^+^ cells from NEC+Teffs (n = 10) and NEC+Tregs (n = 8) groups. All data are from MLN of day 3 NEC induction within NEC+Teffs and NEC+Tregs groups.
(TIF)
