# Supplementary figures and images for: FoxP3+ Regulatory T Cells Attenuate Experimental Necrotizing Enterocolitis
Source: PLoS One. 2013 Dec 18;8(12):e82963. doi: 10.1371/journal.pone.0082963 (PMC3867412; doi:10.1371/journal.pone.0082963)

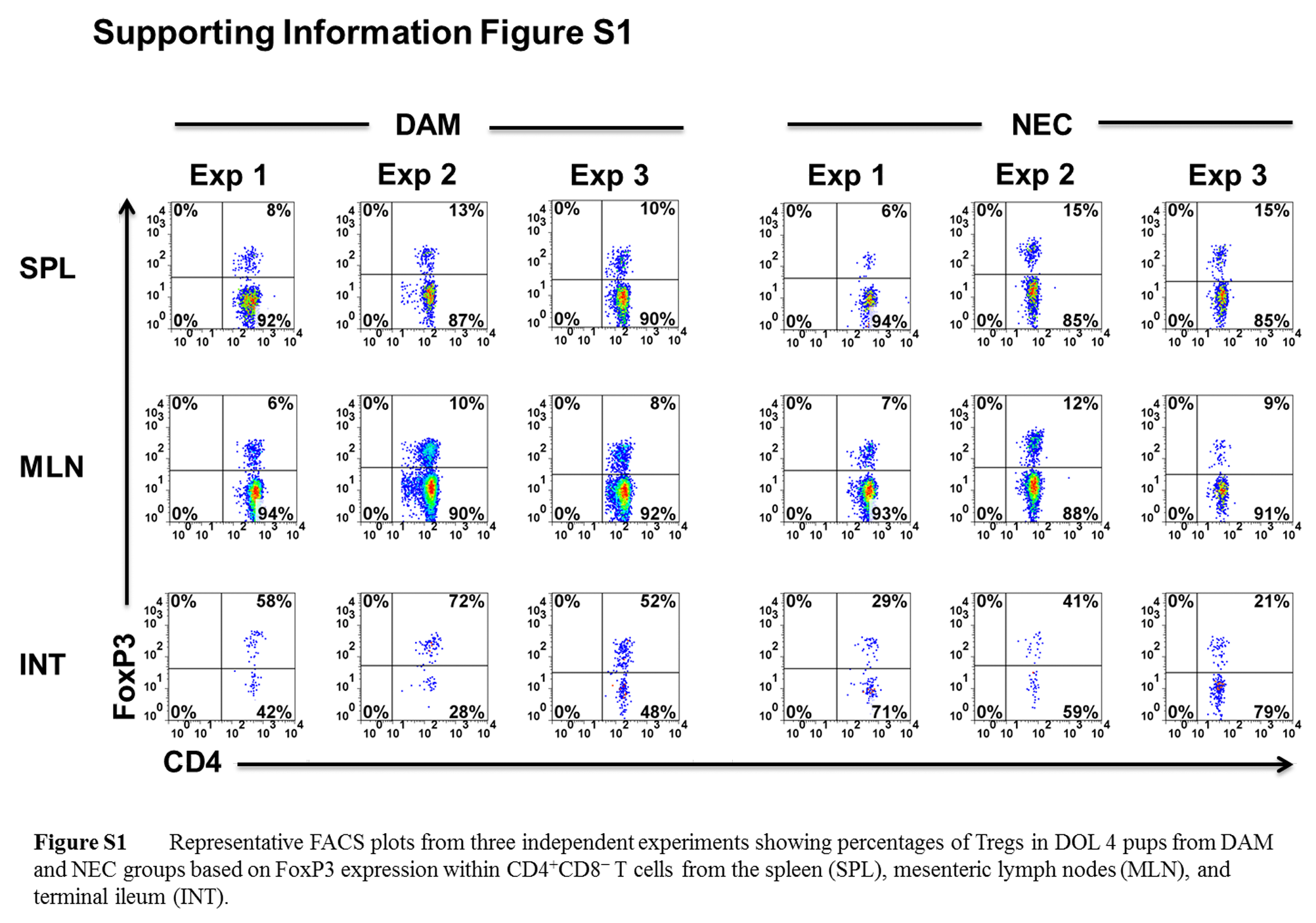

Supplement: Figure S1 — Representative FACS plots from three independent experiments showing percentages of Tregs in DOL 4 pups from DAM and NEC groups based on FoxP3 expression within CD4+CD8− T cells from the spleen (SPL), mesenteric lymph nodes (MLN), and terminal ileum (INT). (TIF) [file pone.0082963.s001.tif]

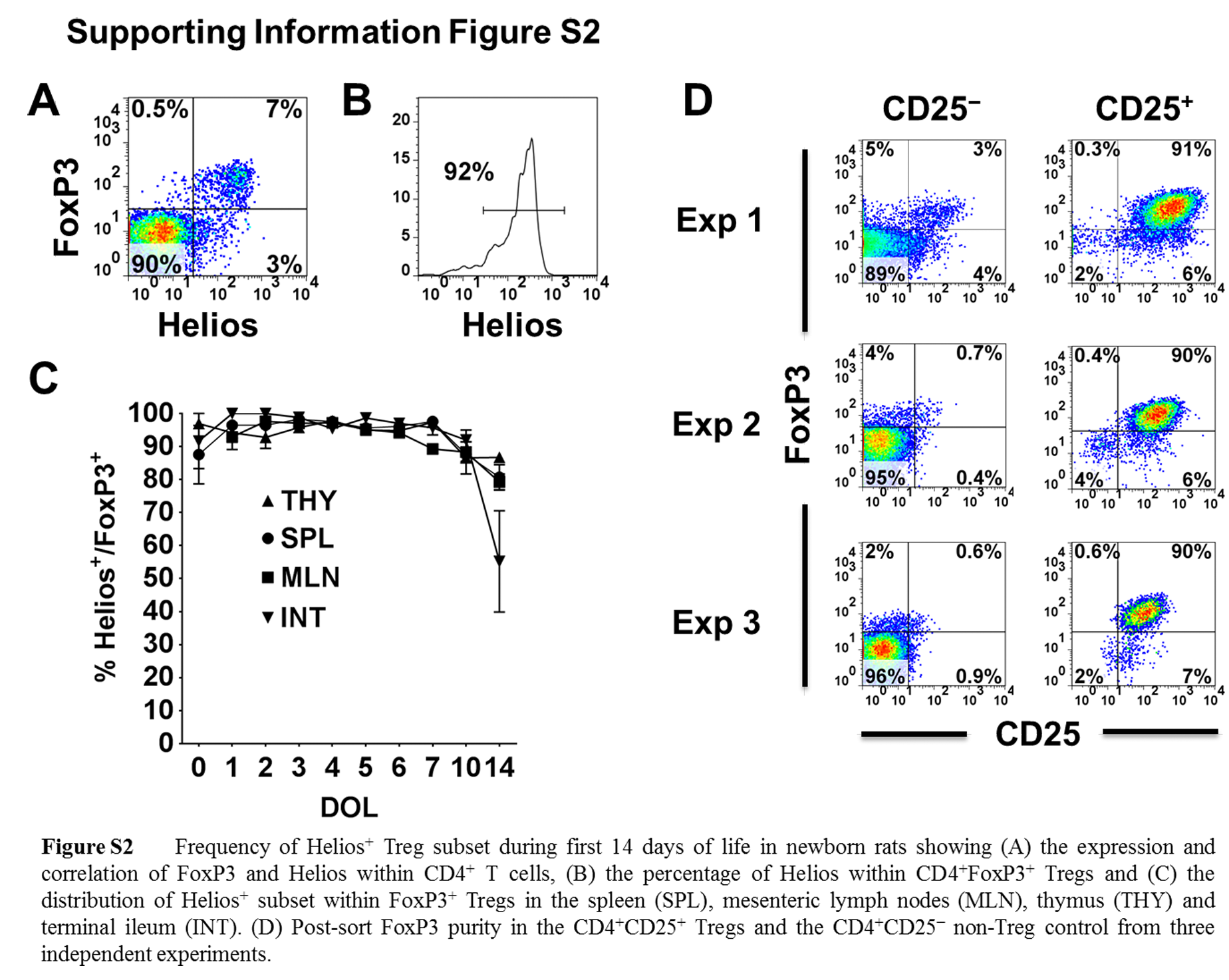

Supplement: Figure S2 — Frequency of Helios+ Treg subset during first 14 days of life in newborn rats showing (A) the expression and correlation of FoxP3 and Helios within CD4+ T cells, (B) the percentage of Helios within CD4+FoxP3+ Tregs and (C) the distribution of Helios+ subset within FoxP3+ Tregs in the spleen (SPL), mesenteric lymph nodes (MLN), thymus (THY) and terminal ileum (INT). (D) Post-sort FoxP3 purity in the CD4+CD25+ Tregs and the CD4+CD25− non-Treg control from three independent experiments. (TIF) [file pone.0082963.s002.tif]

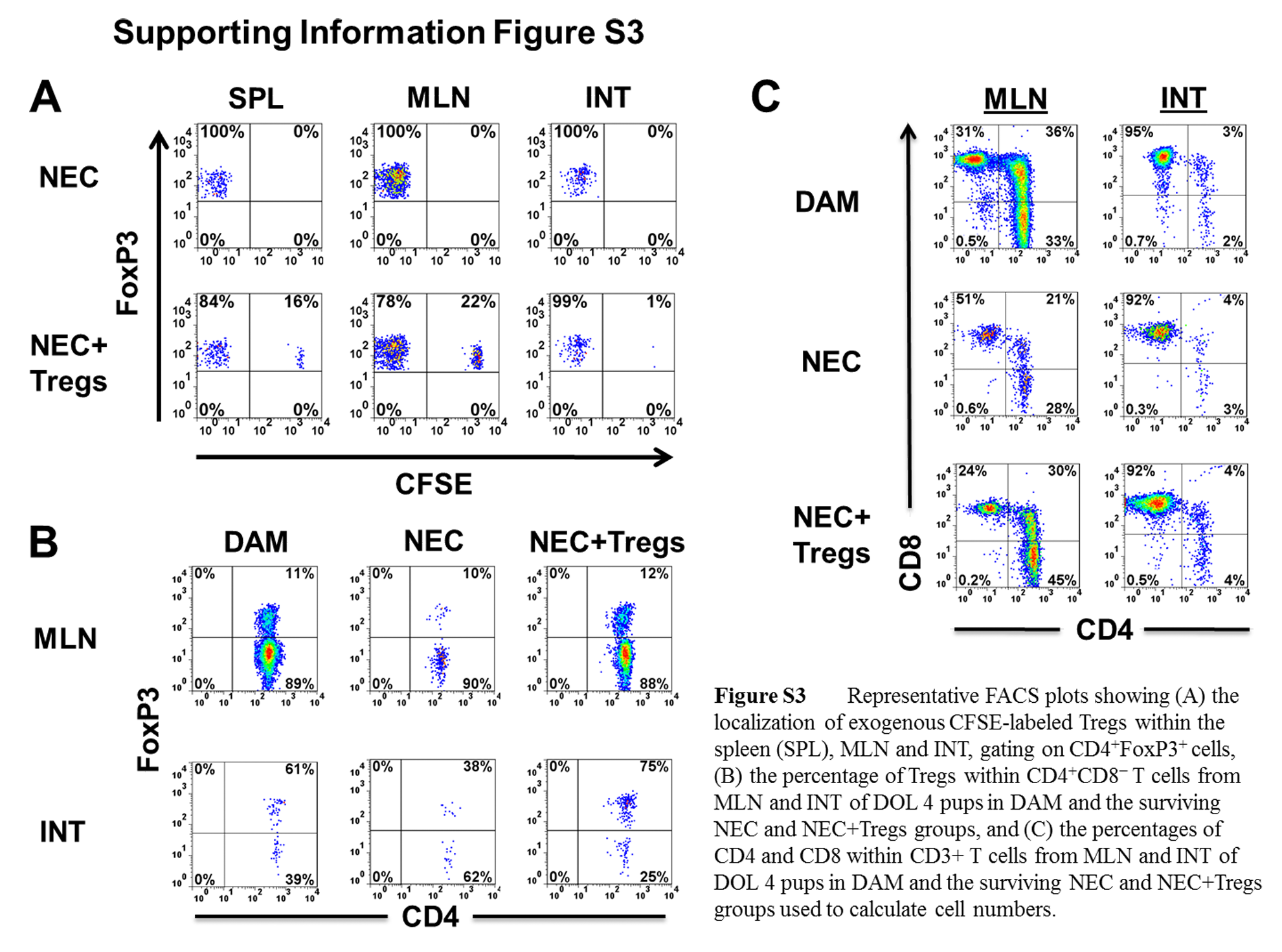

Supplement: Figure S3 — Representative FACS plots showing (A) the localization of exogenous CFSE-labeled Tregs within the spleen (SPL), MLN and INT, gating on CD4+FoxP3+ cells, (B) the percentage of Tregs within CD4+CD8− T cells from MLN and INT of DOL 4 pups in DAM and the surviving NEC and NEC+Tregs groups, and (C) the percentages of CD4 and CD8 within CD3+ T cells from MLN and INT of DOL 4 pups in DAM and the surviving NEC and NEC+Tregs groups used to calculate cell numbers. (TIF) [file pone.0082963.s003.tif]

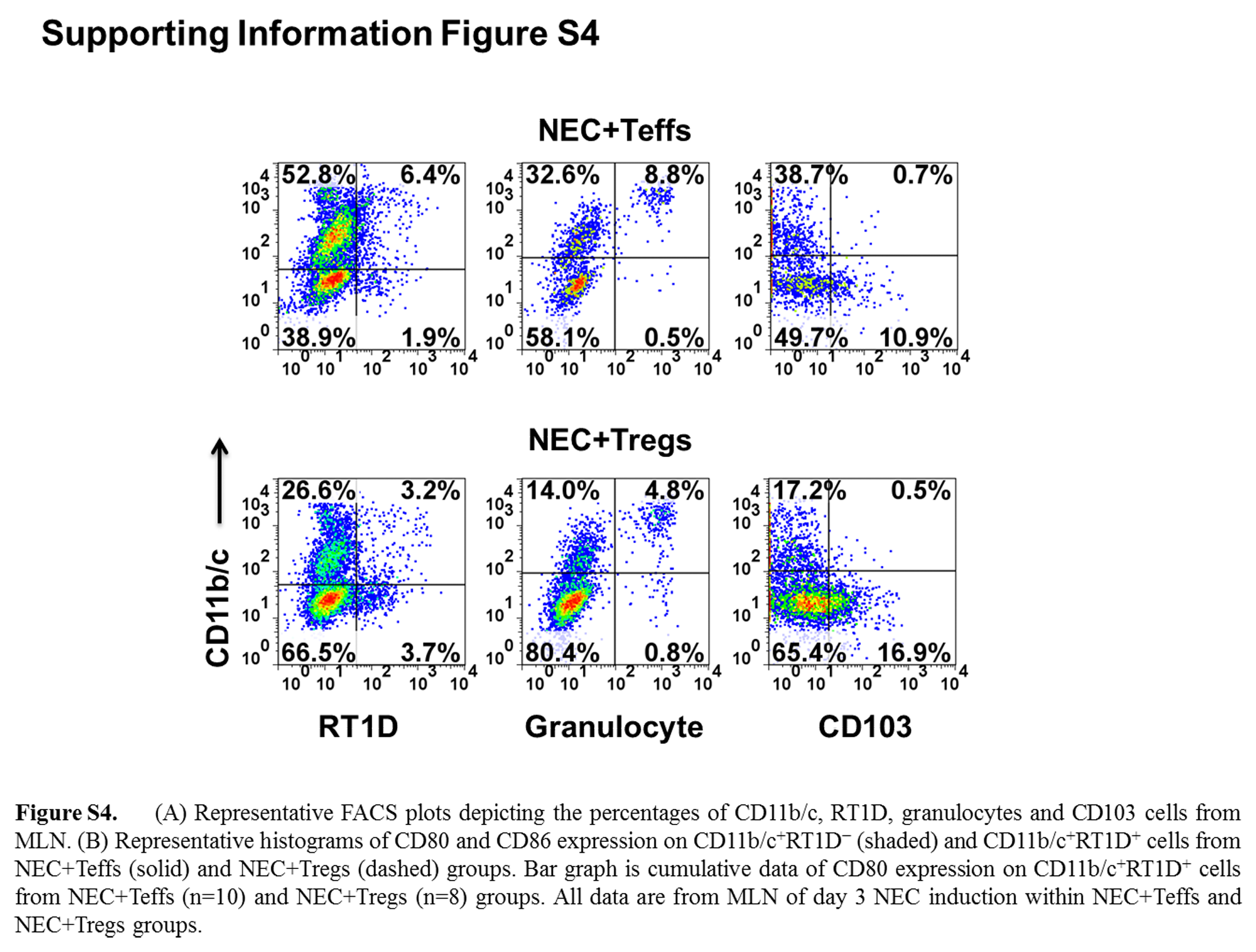

Supplement: Figure S4 — (A) Representative FACS plots depicting the percentages of CD11b/c, RT1D, granulocytes and CD103 cells from MLN. (B) Representative histograms of CD80 and CD86 expression on CD11b/c+RT1D− (shaded) and CD11b/c+RT1D+ cells from NEC+Teffs (solid) and NEC+Tregs (dashed) groups. Bar graph is cumulative data of CD80 expression on CD11b/c+RT1D+ cells from NEC+Teffs (n = 10) and NEC+Tregs (n = 8) groups. All data are from MLN of day 3 NEC induction within NEC+Teffs and NEC+Tregs groups. (TIF) [file pone.0082963.s004.tif]
